# Synergistic effects of applying static magnetic fields and diazepam to improve EEG abnormalities in the pilocarpine epilepsy rat model

**DOI:** 10.1038/s41598-022-26870-z

**Published:** 2023-01-05

**Authors:** Carmen de Labra, Javier Cudeiro, Casto Rivadulla

**Affiliations:** 1 NEUROcom, Centro Interdisciplinar de Química e Bioloxía (CICA), Universidade da Coruña, Rúa as Carballeiras, 15071 A Coruña, Spain; 2 NEUROcom, Facultade de Enfermería e Podoloxía, Universidade da Coruña, Campus de Esteiro, Ferrol, Spain; 3 Instituto de Investigación Biomédica de A Coruña (INIBIC), Complexo Hospitalario Universitario de A Coruña (CHUAC), Sergas, As Xubias, 15006 A Coruña, Spain; 4 NEUROcom, Facultade de Ciencias da Saúde, Universidade da Coruña, Campus de Oza, A Coruña, Spain; 5 Centro de Estimulación Cerebral de Galicia, A Coruña, Spain

**Keywords:** Diseases of the nervous system, Epilepsy

## Abstract

The lithium-pilocarpine rat model is a well-known model of temporal epilepsy. Recently we found that transcranial static magnetic stimulation (tSMS) delay and reduce the signs of EEG in this model. We aim to test the effect of combining the therapeutic action of tSMS and diazepam, a drug used to treat status epilepticus. We induce epilepsy in 12 Sprague–Dawley rats. Animals were classified as “*magnet*” when a magnetic neodymium cylinder was placed over the skull or “*control*” when a stainless-steel replica was used. Diazepam was injected 60-min after the second doses of pilocarpine injection. We found a reduction in the number of spikes/minute for magnet condition compared with sham condition, reaching significance at 60 min after diazepam injection. The Root-Mean-Square shown a significant reduction in magnet animals compared with those receiving diazepam (Tukey’s-test 30 and 60 min after diazepam injection, *p* < 0.01; 40 and 50 min after diazepam injection, *p* < 0.05). Furthermore, the power spectrum analysis shown a reduction in delta, theta, alpha and beta bands, on the diazepam + magnet animals compared to the diazepam + sham group. Analysis of high-frequency oscillations revealed an increased in the ripples due to pilocarpine being reduced by diazepam. Our results demonstrate that application of tSMS previously to diazepam potentiates the effect of the drug by reducing the electroencephalographic pattern associated with epileptiform discharges. We suggest a new synergistic cooperation between pharmacology and neuromodulation as a future treatment for epilepsy.

## Introduction

Epilepsy is a neurological disorder characterized by recurrent seizures, as a result of hypersynchronous excitatory electrical discharges from a group of neurons in the brain. Besides its severity and collateral comorbidities^1^, the epidemiological studies indicate that 6 million people in Europe^2^ and around 70 million people in the world^3^ are affected by this condition. Although the number of antiepileptic drugs (AEDs), including the group of benzodiazepines/barbiturates which interact with GABA-A receptors^4^, have grown in the last 40 years, and 60–70% of patients respond to them, there are still many people who cannot benefit from AEDs. For a number of cases surgery could be the option^5^, but considering that these procedures are not free of risk and complications, finding new treatments is a priority.

Over the past two decades, non-invasive brain stimulation (NIBS) emerged as valuable tool for treatment of neurological and psychiatric disorders^6,7^. In the particular case of epilepsy, the results have been inconclusive probably due to the variability in the stimulation protocols and etiological heterogeneity (for a comprehensive review see^8^). One of the most recently incorporated technique to the NIBS family is transcranial static magnetic stimulation (tSMS)^9^, whose application produces a clear and reproducible reduction of cortical excitability^10–15^. Following this line, in a recent paper we demonstrated that tSMS of moderate intensity (0.5 T) applied alone was able to delay and reduce the characteristic EEG signs of epileptiform discharges, both in rat and monkey^13^.

Given this background and taking into account studies demonstrating the synergistic effect of combination therapy regimens^16^, we wondered if the protective effects we have obtained using tSMS alone could enhance pharmacological strategies, even when the drug is used at very low doses. Based on that, the aim of the present study was to study the putative synergistic effect of combining tSMS with low doses of diazepam, a drug commonly used to treat status epilepticus, in the litio-pilocarpine rat model^17^.

## Materials and methods

All the methods were carried out in compliance with the ARRIVE (Animal Research: Reporting of In Vivo Experiments) guidelines. The study was carried out following the rules of the Physiological Spanish Society, the International Council for Laboratory Animal Science, and the European Union guidelines (No. 2010/63/EU) for the protection of the laboratory animals used for scientific purposes. The ethics committee for animal research of the University Hospital of A Coruña approved the experimental protocol (P090). All efforts were made to minimize animal suffering and the number of animals used.

The animals used in this study were 12 male and female Sprague–Dawley rats, 2–3 months old. Animals were housed in standard cages on a 12/12-h light/dark cycle, having free access to food and tap water.

### Electrode implantation for Electrocorticogram recording

Animals were introduced in a cage for anesthesia induction (5% sevofluorane). Once anesthetized, rats were placed in a stereotaxic frame and anesthesia was reduced to 3.5%. Once the animal was stabilized, the skin was opened, and the connective tissue removed until lambda and bregma landmarks were visible. Using a hand drill, two small craniotomies were made in the skull of each rat for the placement of two electrodes (stainless screws, 1.2-mm diameter, 4-mm length) to record electrocorticogram signals (ECoG). The active electrode was placed on the area of the right somatosensory cortex (AP − 1.5 mm from Bregma, L 2 mm from the midline) and the reference electrode was placed on the left primary visual cortex (AP -6.3 mm, L 3.5 mm).

### Data acquisition

Continuous ECoG recordings started immediately after the surgery (at this stage the anesthesia level was set at 1.5–2% of sevofluorane). The ECoG signal was amplified using an A-M system differential amplifier (Model 1700 A-M System, LLC, Calsborg, WA, USA), band-pass filtered (gain 1000, range 1 Hz–500 Hz), digitized at 20 kHz (1401 CED A/D convertor card; Cambridge Electronic Design, UK) and stored (Spike 2 software; Cambridge Electronic Design, UK) in a PC for online checking and posterior analysis. The animals remained anaesthetized throughout the experiment.

### Induction of status epilepticus and antiepileptic drug administration

**Figure 1:**
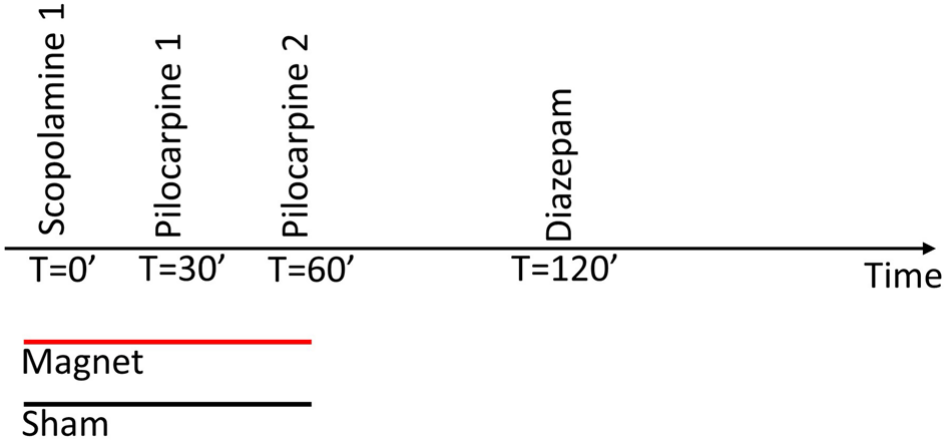
Schematic diagram showing the time schedule for the magnet and drug administration protocol during the recording session: T = 0 scopolamine (1 mg/kg), T = 30 pilocarpine (20 mg/kg), T = 60 pilocarpine (20 mg/kg), T = 120 Diazepam (1.25 mg/kg), all drugs were i.p. administered. Magnet or Sham were applied for 1 h, starting at T = 0.

We used the lithium-pilocarpine model to induce epilepsy^18^. 24 h before the surgery procedure described above the animals were intraperitoneally (i.p.) injected with 127 mg/kg of LiCl, and returned to their cages. Immediately after the surgery (always under anesthesia) we checked the quality of the ECoG signal, and after thirty minutes of stable recording, scopolamine (1 mg/kg) was injected (i.p.). After thirty minutes of scopolamine, two doses of pilocarpine (20 mg/kg each), thirty minutes apart, were administered (i.p.). Finally, diazepam (1.25 mg/kg) was i.p. injected 60 min after the second dose of pilocarpine. A schematic diagram showing the protocol timeline is illustrated in Fig. 1. In order to stablish the appropriate dose a preliminary experiment was carried out (n = 4) using 10.00, 2.50, 1.25 and 0.75 mg/kg of diazepam. 10.00 and 2.50 mg/kg doses of diazepam produced a practically total suppression of the oscillatory activity with long periods of isoelectric activity and with clear affectation of the respiratory function, and 0.75 mg/kg had not a noticeable effect on a visual inspection (data not shown). These pilot experiments showed us that the best doses for testing our hypothesis was 1.25 mg/kg of diazepam, as it was the lowest doses at which a pharmacological effect could be seen.

### Application of tSMS

Animals were randomly classified into two groups: One to be exposed to a magnetic field (Magnet group) and the other to be exposed to a stainless-steel replica (Sham group) without magnetic activity. We used a cylindrical nickel-plated (Ni–Cu–Ni) NdFeB magnet with a 45-mm diameter and a 30-mm thickness that weighed 360 g (NEUREK S.L., Toledo Spain). The maximum amount of magnetic energy stored in this magnet was 45 MGOe, with a nominal strength of 765 N (78 kg). Previous experiments, including actual measurements and computer models^19–22^ showed with a remarkable consistence a magnetic field of 0.5 T on the magnet surface and ∼0.3 T at 1 cm. A nonmagnetic replica of identical appearance and weight (i.e., indistinguishable from the magnet) was used for the sham stimulation (NEUREK S.L., Toledo Spain).

The magnet (or the replica) was placed on the rat's head (in contact with the skull) coinciding with the scopolamine injection, it was maintained for one hour and was removed when the second dose of pilocarpine was administered, hence the magnet did not overlap in time with diazepam administration. The magnet was placed with the field lines perpendicular to the cortical surface.

### ECoG and data analysis

Data analyses were performed with Spike2 software (Cambridge Electronic Design, v8.03) and home-made routines in MATLAB.

The raw ECoG signal was downsampled to 1 kHz and recorded continuously from the beginning of the experiments. Every 30 min, an epoch of 60 s was automatically selected and analyzed in terms of epileptic spikes (normalized peak per minute) and root mean square (RMS), using specific tools included in the spike2 software. Epileptic spikes, considered as sudden change of the ECoG signal, have been long documented as a marker of the epileptic processes happening in the brain^23^. RMS is a common method to estimate the ECOG amplitude. For a given set of values, RMS is the square root of the arithmetic mean of the squares of these values. In the case of a DC signal or a non-sinusoidal signal, RMS provides an estimation of the averaged amplitude^24^.

We performed an analysis of the power spectral density (PSD) at the different experimental conditions, in four frequency bands separately: Delta (0 to 4 Hz), theta (4 to 8 Hz), alpha (8 to 12 Hz) and beta (12 to 30 Hz).

Finally, we also analyzed high frequency oscillations (HFOs). Raw ECoG signals were bandpass filtered between 80-200 Hz. In this frequency of 80-200 Hz, an oscillation with 4 or more consecutive peaks and with an amplitude higher than 3 SD of the mean was considered as a ripple^25^.

Statistical analyses were performed using RStudio (version 2022.07.1) software. Values were expressed as the mean ± standard error. Analysis of peaks per minute, RMS, PSD at the different frequency bands, and ripples, were analyzed using a repeated-measures analysis of variance (ANOVA). The degrees of freedom were corrected (if needed) with the Greenhouse–Geisser coefficient. Factors included CONDITION (sham and magnet) and TIME_drug_injection (baseline, prediazepam and postdiazepam). We used Tukey’s multiple comparison test for post hoc analysis. All results were considered significant at *p* < 0.05.

## Results

**Figure 2:**
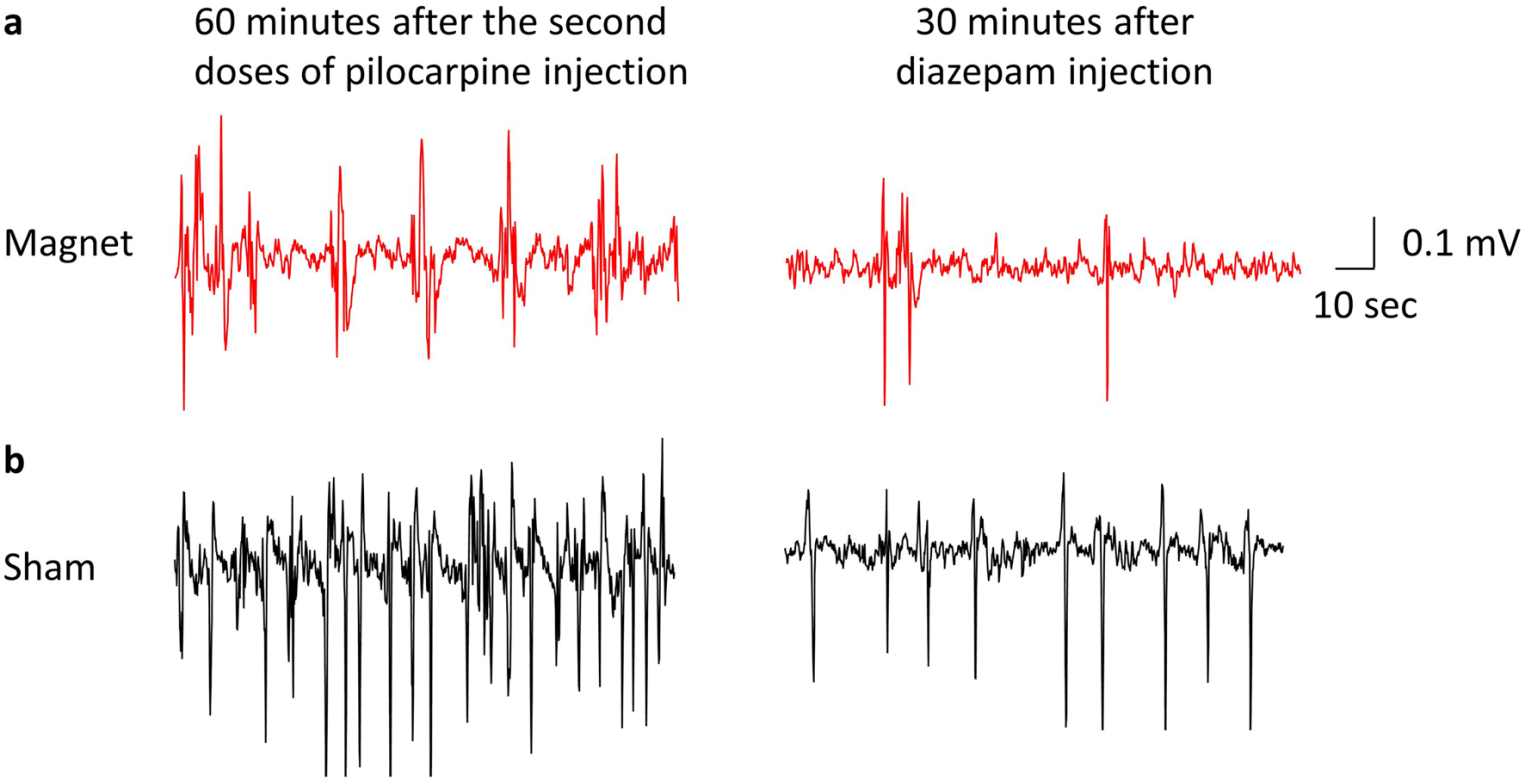
A 10 s sample EEG of two animals obtained 60 min after the second dose of pilocarpine (left) and 30 min after diazepam injection (right). Red traces represent the animal exposed to the real magnet (**a**). Black traces represent the sham (**b**).

**Figure 3:**
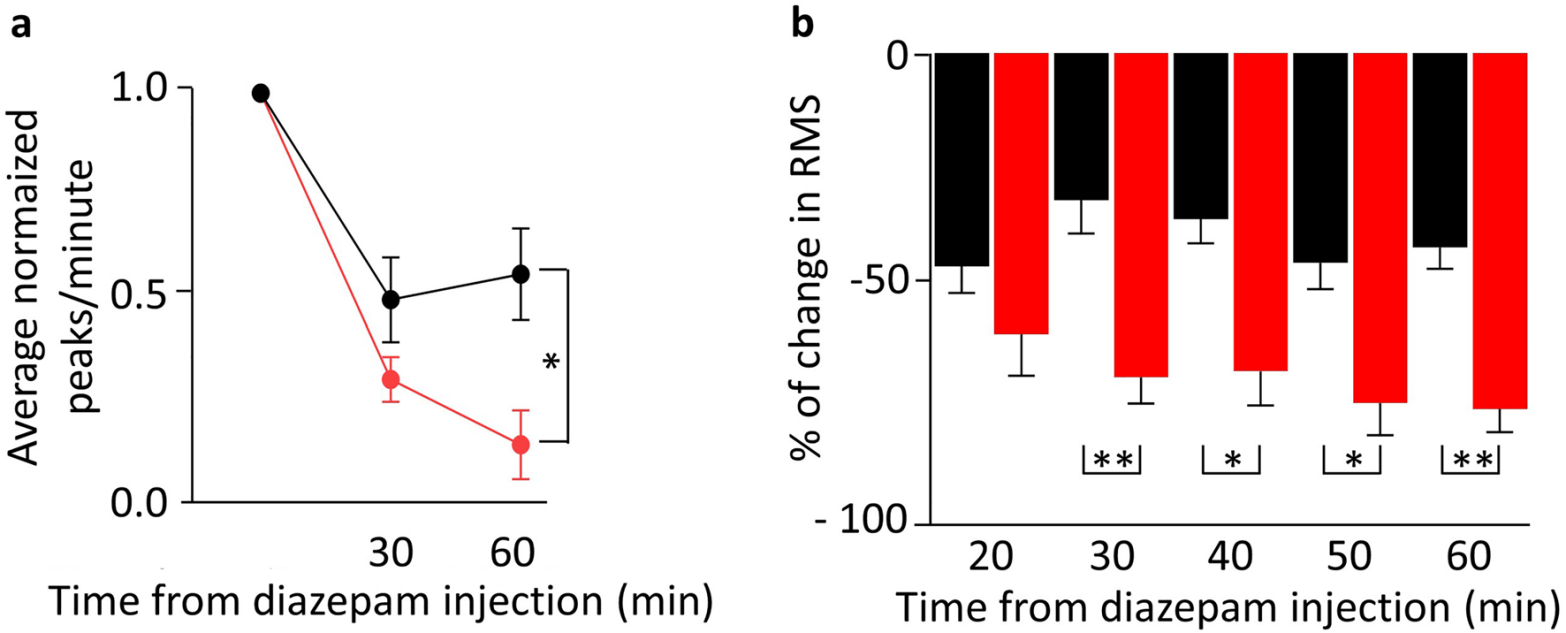
(**a**) Average number of spikes at different time points after diazepam injection (doses 1.25 mg/kg, i.p.). Black dots represent data obtained from animals exposed to sham whereas red dots represent animals exposed to real magnet. Note that the data is normalized. (**b**) Bar histogram representing the percentage of reduction in the spontaneous Root Mean Square (RMS) at different time points after diazepam injection (doses 1.25 mg/kg, i.p.). Black bars, sham rats; red bars, magnet rats. In both cases (**a**, **b**), data represented 5 min epochs.

Figure 2 shows two ECoG samples obtained from two different animals, one with the magnet (Fig. 2a), and one with the replica (Fig. 2b), one hour after the second dose of pilocarpine (left panel), and thirty minutes after diazepam injection (right panel). As we have shown previously^13^ the magnet prevented the appearance of epileptic signs in the ECoG, this is, after pilocarpine injection the animal with the real magnet continued to show the up and down states characteristic of the anesthesia while the sham shows an altered ECoG with a spike pattern. We have considered the criterion to define the abnormal pattern of spikes a discharge that last at least 0.3 s, with an amplitude at least two times higher than the baseline. It should be noted that while in our previous manuscript the magnet was placed at the time of the first pilocarpine dose, here it was placed at the same time of the scopolamine injection. Diazepam, dispensed at a dose of 1.25 mg/kg (i.p.), was administered one hour after the second pilocarpine injection, and it is clear from the recording that thirty minutes after diazepam injection, there was a clear reduction in the number of ECoG spikes in both animals, being more pronounced in the animal with the magnet (for a population analysis, see Fig. 3).

To assess the severity of the induced status epilepticus and the magnitude of the antiepileptic effect obtained with our manipulations, we measured the number of spikes/minute and the RMS. Regarding the number of spikes/minute, a repeated measures ANOVA revealed both, a significant interaction effect between group (sham and magnet) and time of drug injection (baseline, prediazepam and postdiazepam) (F (1, 14) = 5.039, *p* = 0.041), and also significant effect between groups (sham and magnet), being reduced significatively the number of spikes in the magnet condition (F (1, 14) = 6.535, *p* = 0.023). Comparison analysis revealed significant differences between groups, such that spikes/minute for the magnet condition at 60 min after diazepam injection were significantly reduced than for the sham condition at 60 min after diazepam injection (pairwise comparison with Tukey’s test, *p* < 0.01). Figure 3b shows the temporal evolution of the diazepam plus magnet (magnet animals) and the diazepam effect (sham animals) on the RMS of spontaneous ECoG activity, and as it can be seen, the RMS showed a greater reduction in the magnet group compared with those animals that only received the diazepam dose (pairwise comparison with Tukey’s test, 30 and 60 min after diazepam injection, *p* < 0.01; 40 and 50 min after diazepam injection, *p* < 0.05). Interestingly, as it is shown in Fig. 3a, while the effect mediated only by diazepam seems to reach the maximum about 30 min after the injection and keeps stable, on those animals pre-treated with the magnet the effect is also clear 30 min after the diazepam, but is even greater at 60 min since the effect continues to grow in the group exposed to the real magnet.

**Figure 4:**
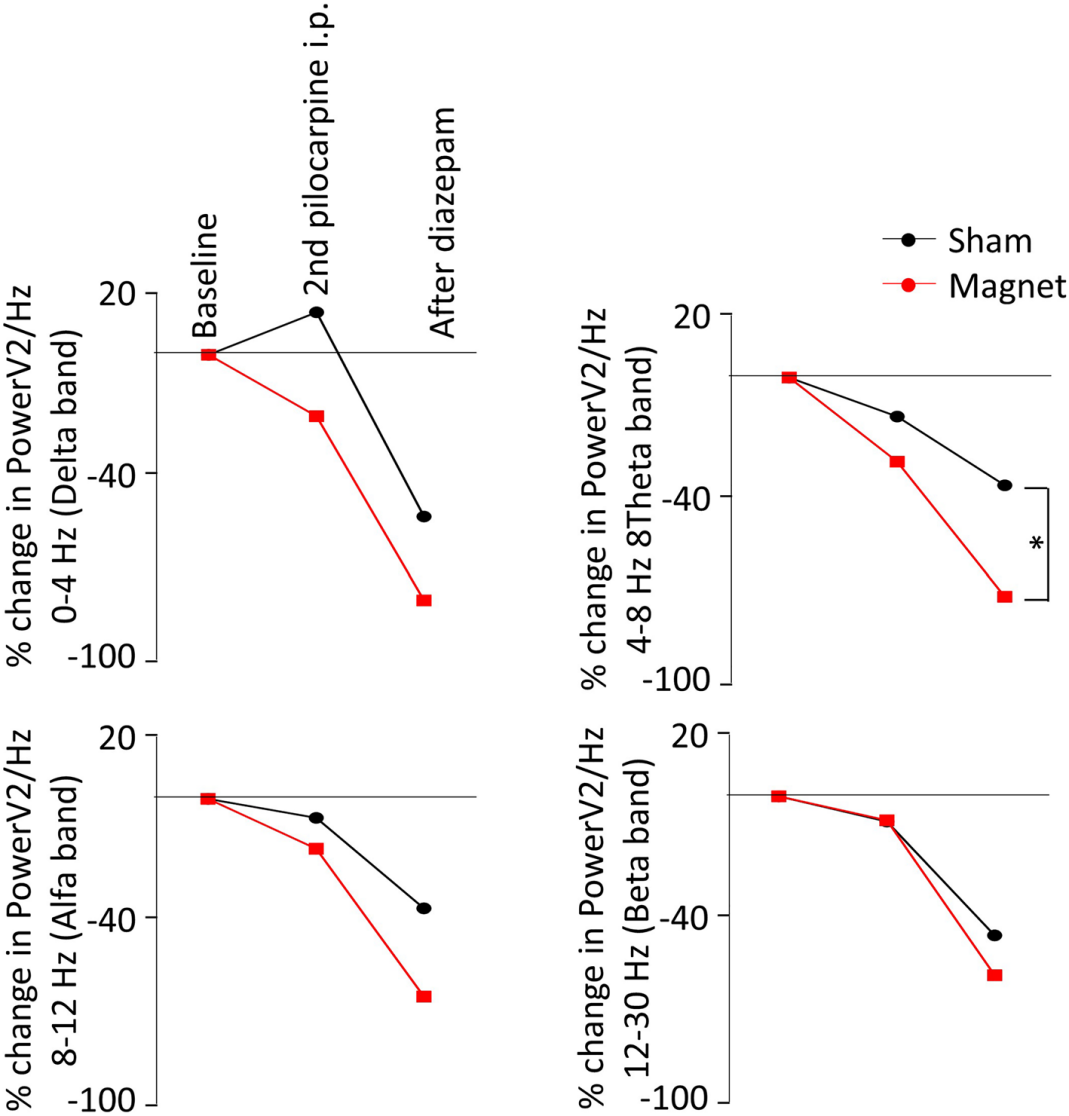
(**a**) Power averaged across frequencies are averaged values from 8 pair of animals (diazepam dose 1.25 mg/kg). Significant differences between Sham and Magnet *p < 0.05. Black, sham rats; red, magnet rats.

We also calculated the power of the ECoG activity, as it is a well stablish method for processing and quantifying the ECoG signal. Bands for delta (0–4 Hz), theta (4–8 Hz), alpha (8–12 Hz), and beta (12–30 Hz) rhythms were obtained averaging three one-hour period epochs, one obtained from baseline, other during the period of the second dose of pilocarpine injection, and the last one after diazepam injection. As it is shown in the Fig. 4, compared to the baseline situation, the power spectrum analysis showed a reduction in all the analysed bands, being this reduction more pronounced in the magnet animals. It is important to note that the reduction becomes bigger and more evident for the magnet animals after diazepam injection, reaching significance that difference in the theta band (pairwise comparison with Tukey’s test, *p* < 0.05).

**Figure 5:**
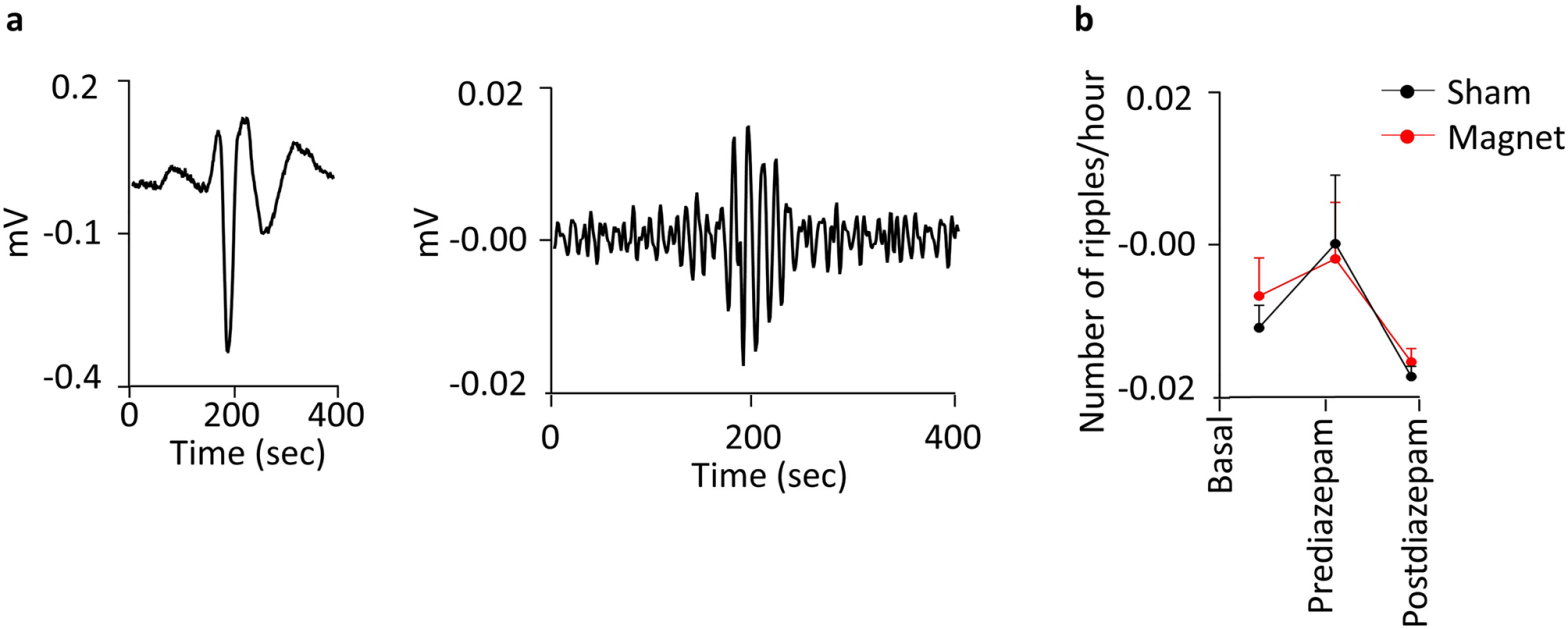
(**a**) Representative recordings from a pilocarpine-treated rat showing the raw ECoG (left) and a ripple (right) (**b**) Number of ripples per hour averaged on 8 pair of animals (diazepam dose 1.25 mg/kg). No significant differences were observed between magnet and sham.

Analysis of high-frequency oscillations, as ripples, could be seen in Fig. 5. In our recordings, ripples were evident after filtering the signal, a representative sample is shown in Fig. 5a (see methods) and were present before and during the seizures^25^. The ripples increased due to the effect of pilocarpine and were reduced by diazepam, however the magnet did not produce any effect on them, Fig. 5b. The mean frequency of the ripples in the different conditions was also analysed. The mean values obtained at baseline, pre and post diazepam, with the sham replica and with the magnet were respectively, for the sham condition 95.70 ± 7.38, 94.62 ± 3.43, 94.62 ± 6.60, and for the magnet condition 90.82 ± 3.20, 88.11 ± 2.17, 85.94 ± 0.00, and according to statistics there were not significant differences.

## Discussion

Noninvasive brain stimulation techniques have recently emerged as useful tools for neuroscience research, clinical evaluation and treatment of various neurological and psychiatric conditions, among them, epilepsy. Based on previous data from our lab, the present study was designed to investigate whether tSMS -a novel NIBS technique- combined with low doses of diazepam can produce a synergistic effect modulating the EEG observed abnormalities in the pilocarpine epilepsy rat model. The idea was straightforward: Develop a combined treatment that allow effective control of epileptic seizures, minimizing the pharmacological doses used; a polytherapy with a single drug (at low doses) which allows reducing the danger of the addition of pharmacological side effects^26^. tSMS has, until date, no demonstrable side effects^27^.

We used the lithium-pilocarpine model of epilepsy in the anaesthetized rat because it is very reliable and produces recurrent seizures easily recorded with EEG^28,29^, which makes it a very suitable model to carry out pharmacological experiments such as the ones we have used in combination with neuromodulation. It does, however, have a limitation. It does not allow to study the behavior of the animals, which could have been useful to establish correlations between the different bands of the EEG and different behavioral stages^30^. However, if we consider our work as a proof of concept to demonstrate the synergistic effect between neuromodulation and pharmacology for the control of seizures, we believe that the experimental design is valid.

Our study yielded four main findings: (i) It is the confirmation that in this rat model of epilepsy, tSMS of moderate intensity prevents the appearance of epileptic signs in the EEG^13^. The magnet is able to produce this effect by reducing the cortical excitability altered in epilepsy. This effect, clear and reproducible, has been revealed by mean of electrophysiological techniques, ranging from single neuron recordings to evoked potentials, but it has also been observed through behavioral correlates. For example, when the magnet was applied to the visual cortex of behaving monkeys or humans, it produced a transient scotoma and a consequent alteration of the visual detection task^10–12^.

(ii) Our data demonstrate a synergistic action between magnetic fields and diazepam in reducing the severity of the epileptiform discharges, measured by the number of spikes and RMS of the ECoG, as compared to the sham-treated animals. Figure 3 shows this effect at diazepam dose of 1.25 mg/kg. To achieve cessation of acute epileptic signs in this model, usual doses are in the range of 5 to 10 mg/kg, 4 to 8 times higher than the ones we used in our experiments^31^.

Our results agree with previous research done in black Swiss mice, where audiogenic epileptic seizures treated with static magnetic fields and phenytoin produce a robust anticonvulsant effect^32^. Furthermore, in this manuscript we demonstrate that application of tSMS was able to develop a protective effect over time; it should be noted that the magnet was present during the pharmacological induction of epileptic status, but it was removed 1 h before the diazepam injection.

We do not know the mechanisms that mediate the effects of tSMS, but it has been postulated that it could affect voltage dependent calcium channels^33^, or that it could mechanically change membrane properties affecting to potassium channels^34^. Whatever the mechanism and whether or not it is more selective on a specific neuronal type (e.g. inhibitory interneurons/excitatory cells), what is well established is that, as a global outcome, it produces a reduction in cortical excitability. Diazepam, on the other hand, acts increasing GABAergic activity, and hence increasing the weight of the inhibition, furthermore, Diazepam could also inhibit voltage dependent sodium channels^35,36^ and voltage dependent calcium channels^37^, hence some mechanisms of action might be similar for tSMS and Diazepan while others are more specific for each of them.

An interesting aspect refers to the temporal course of the effects observed after tSMS. In previous studies it has been shown that some physiological measurements as motor evoked potentials or visual detection^9,11^ were completely recovered 20 or 30 min after being affected by magnet application. Here we have shown that after the injection of diazepam, 1 h apart from removing the magnet, there were still differences between the animals exposed to the magnet and those that were exposed to the sham, indicating that the effect of the magnet was maintained over time. This suggests the possible existence of a temporal window after the action of the magnet in which the action of other interventions is favored. These aspects will be the subject of future experiments.

(iii) Power spectral analysis, a measure related with the severity of the epilepsy^38^ shows a decrease in all bands studied (delta, theta, alfa, and beta) after applying the diazepam. This effect tends to be higher in those animals previously treated with magnet, reaching significant values at theta band. The reduction we observed in these bands agrees with other authors^38^ that found that any increase is associated to a greater number of seizures and the other way around. Our study showed that the theta band was the most affected frequency, and it fits well with proposals from other authors, namely, that the theta band is the preferred frequency for investigating the brain-network dysfunction^26^, and that this frequency band is a good epileptic marker^39^.

(iv) The results obtained with ripple analysis were somewhat surprising. The observed rate of ripples was similar between magnet and sham conditions, during the three different periods studied: baseline, prediazepam, and postdiazepam. For both conditions (sham and magnet), ripples were increased as a consequence of pilocarpine and decreased as a result of diazepam injection. We have not been able to verify any effect on them produced by the application of the magnet.

It has been demonstrated, using 4-aminopyridine treated rats, that seizures leading to status epilepticus show a higher number of ripples when compared to seizures leading to isolated seizures. As ripples appear to originate in the principal cells of the hippocampus, and inhibition of these neurons delays the initiation of status epilepticus^40^, our result suggest that diazepam could act on the firing of these hippocampal excitatory neurons and hence inhibiting the electrographic sings of epilepsy. However, the magnet did not affect ripples; it could be an indication of the limits of action of the magnetic field, since the intensity is highly reduced with the distance^19^.

## Conclusions

In summary, our results demonstrate that application of tSMS previously to diazepam potentiates the effect of the drug by reducing the electroencephalographic pattern associated with epilepsy. There are several questions that have to be addressed in future experiments in order to establish a new antiepileptic protocol from our data: Optimal time interval between tSMS and drug injection, different dose/response relationships, increase or decrease in tSMS time and dose of diazepam. Even taking all this into account, we believe that our results open the door to a new kind of cooperation between pharmacology and neuromodulation as a future treatment for epilepsy.

## Data Availability

All data, and materials used in the analysis will be made available on request for proposals that set out to achieve aims specified in a methodologically and scientifically sound protocol. Applications to access data can be sent to c.labra@udc.es. Data access will be used for non-commercial, academic, and research use only.
